# Translating Science Into Practice and Making It Stick: System-Level Approaches

**DOI:** 10.1093/geroni/igab046.2062

**Published:** 2021-12-17

**Authors:** Laura Wray, Kim Curyto, Jennifer Sullivan

**Affiliations:** 1 VA Center for Integrated Healthcare, Buffalo, New York, United States; 2 VA Western New York Healthcare System, Batavia, New York, United States; 3 VA Providence Medical Center LTSS COIN and Brown University, Providence, Rhode Island, United States

## Abstract

The delay between establishing evidence-based practice and implementing this evidence base is well documented. This presentation will focus on the application of implementation science principles to real-world clinical programs. A VA priority is to implement evidence-based practice for management of DBD in CLCs. Key implementation science concepts will be introduced, along with a description of how these conceptual models facilitate application of roll-out and sustainment of complex evidence-based interventions. Conceptual frameworks that contributed to intervention selection and facilitation of STAR-VA implementation, including the Consolidated Framework for Implementation Research (CFIR) and Knowledge Reservoirs (KR) framework, and their application in health care practice, will be discussed. The CFIR Expert Recommendation for Implementing Change (ERIC) Mapping Tool will be introduced as useful to identify strategies that address barriers to sustaining implementation. Attendees will be provided with resources to support implementation and sustainment efforts.

